# Impact of a Participatory Intervention with Women’s Groups on Psychological Distress among Mothers in Rural Bangladesh: Secondary Analysis of a Cluster-Randomised Controlled Trial

**DOI:** 10.1371/journal.pone.0110697

**Published:** 2014-10-16

**Authors:** Kelly Clarke, Kishwar Azad, Abdul Kuddus, Sanjit Shaha, Tasmin Nahar, Bedowra Haq Aumon, Mohammed Munir Hossen, James Beard, Anthony Costello, Tanja A. J. Houweling, Audrey Prost, Edward Fottrell

**Affiliations:** 1 Institute for Global Health, University College London, London, United Kingdom; 2 Perinatal Care Project, Diabetic Association of Bangladesh, Dhaka, Bangladesh; 3 Department of Public Health, Erasmus MC University Medical Center Rotterdam, Rotterdam, The Netherlands; Medical University of Vienna, Austria

## Abstract

**Background:**

Perinatal common mental disorders (PCMDs) are a major cause of disability among women and disproportionately affect lower income countries. Interventions to address PCMDs are urgently needed in these settings, and group-based and peer-led approaches are potential strategies to increase access to mental health interventions. Participatory women’s health groups led by local women previously reduced postpartum psychological distress in eastern India. We assessed the effect of a similar intervention on postpartum psychological distress in rural Bangladesh.

**Method:**

We conducted a secondary analysis of data from a cluster-randomised controlled trial with 18 clusters and an estimated population of 532,996. Nine clusters received an intervention comprising monthly meetings during which women’s groups worked through a participatory learning and action cycle to develop strategies for improving women’s and children’s health. There was one group for every 309 individuals in the population, 810 groups in total. Mothers in nine control clusters had access to usual perinatal care. Postpartum psychological distress was measured with the 20-item Self Reporting Questionnaire (SRQ-20) between six and 52 weeks after delivery, during the months of January to April, in 2010 and 2011.

**Results:**

We analysed outcomes for 6275 mothers. Although the cluster mean SRQ-20 score was lower in the intervention arm (mean 5.2, standard deviation 1.8) compared to control (5.3, 1.2), the difference was not significant (β 1.44, 95% CI 0.28, 3.08).

**Conclusions:**

Despite promising results in India, participatory women’s groups focused on women’s and children’s health had no significant effect on postpartum psychological distress in rural Bangladesh.

## Introduction

Perinatal common mental disorders (PCMDs) are defined as depressive, anxiety, panic and somatic disorders occurring in pregnancy or the postpartum period [1]. PCMDs are a major cause of disability among women, and depression is a strong predictor of suicide [2]–[5]. PCMDs also have adverse consequences for children’s growth and development, and disrupt the mother-infant bond [6]–[8]. Women in low and lower middle-income countries are disproportionately affected, with 16% (95% Confidence Interval [CI] 15.0–16.8%) experiencing a PCMD during pregnancy and 20% (95% CI 19.2–20.6%) in the postpartum period [9]. However, to date there are few trials of interventions for PCMDs in these countries [10], [11]. Interventions delivered by non-mental health specialists and integrated into existing maternal and child health programmes are needed [10]–[12].

Women’s groups practising participatory learning and action to address women’s, newborn and child health problems have been implemented in several African and south Asian settings [13]. Groups are facilitated by lay women or community health volunteers and are open to all community members, though they target women of reproductive age. Although groups do not explicitly address PCMDs, a recent meta-analysis showed that exposure to groups is associated with a 37% reduction in neonatal mortality (Odds Ratio [OR] 0.63 95% Confidence Interval [CI] 0.32, 0.94) a key predictor of PCMDs [13]. Groups may also reduce maternal complications during the perinatal period and neonatal deaths, improve social support and problem-solving skills, and are therefore a potential strategy to improve maternal mental health [14], [15]. In rural eastern India exposure to groups reduced moderate postpartum psychological distress, a proxy for PCMDs, by 57% (OR 0.43 95% CI 0.23, 0.80) within geographical clusters that had received the intervention, albeit only in the third year of the trial [15].

Population coverage, as well as the proportion of pregnant women attending groups, predict the effect size of women’s groups on neonatal and maternal mortality [13]. In rural eastern India, population coverage was 468 per group and the proportion of pregnant women attending at least one group meeting was 55% in the final year of intervention, during which time the reduction in moderate postpartum psychological distress was detected [14]. In rural Bangladesh women’s groups have been evaluated in two trials. In the first trial (2005–2007) coverage was 1414 per group, only 3% of pregnant women attended groups (477/15,695) and there was no impact on neonatal mortality [16]. In the second trial (2009–2011) coverage increased to 309 per group, attendance among pregnant women increased to 36% (3326/9109) and neonatal mortality was reduced by 38% (Risk Ratio [RR] 0.62 95% CI 0.43, 0.89) [17]. In Bangladesh there is a high prevalence of PCMDs ranging from 22 to 52%, and a lack of mental health resources in rural areas [18]–[20]. In light of proven mental health benefits of women’s groups in rural eastern India, and using data from the second Bangladeshi trial, we aimed to assess the impact of groups on postpartum psychological distress in rural Bangladesh.

## Methods

### Ethics statement

The trial was approved by the Ethical Review Committee of the Diabetic Association of Bangladesh and by University College London Research Ethics Committee (ID number: 1488/001), and registered as ISRCTN01805825. Permission for the trial was obtained from community leaders. The consent process, approved by the ethics committees, involved briefing respondents about the trial aims, the interview process and data storage and usage, and respondents were free to decline participation. We sought verbal, as opposed to written, consent from respondents because many of them were illiterate. Consent was documented by the interviewer at the time of interview and subsequently entered into the trial database. Respondents were made aware of appropriate healthcare facilities if they or their children were ill.

### Study setting and design

We conducted a secondary analysis of a cluster-randomised controlled trial of participatory women’s groups to address women’s, newborn and child health problems in rural Bangladesh. Neonatal mortality was the primary outcome and postpartum psychological distress, a proxy for PCMDs, was an additional outcome. The full design of the trial has been reported elsewhere [17], [21]. Briefly, January to December 2008 was the baseline period and the trial ran from 1^st^ January 2009 to 30^th^ June 2011. The trial was located in Moulvibazar, Faridpur and Bogra districts, which were purposively selected because of the presence of Diabetic Association of Bangladesh (BADAS) programmes, and to represent the geographically diversity of rural Bangladesh. In each district two upazillas (sub-districts) and three unions per upazilla were sampled on the basis of being accessible from BADAS district headquarters but having limited access to perinatal health care facilities. Unions comprise a group of adjacent villages, are the lowest administrative unit in Bangladesh and were the unit of clustering in the trial.

The six unions in each district (18 unions in total) were randomly allocated to both intervention and control arms by drawing folded papers from a bottle using a pre-specified allocation sequence. The estimated population size in the study unions was 532,996 people, in 117,914 households [17]. Around 80% of the population is Muslim, and the rest are mainly Hindu. Around half of the women who recently delivered never went to school or only attended primary education. Baseline neonatal (38/1000) and pregnancy-related (188/100000) mortality rates in the study districts were high [17]. In Moulvibazar there are several tea garden estates. Workers on these estates (tea garden residents) are mainly of Indian descent and Hindu, since their ancestors were brought from India by former British rulers [22], [23]. They tend to be socioeconomically disadvantaged compared to non-tea garden residents.

### Intervention and control arm activities

Women’s groups comprised 162 ‘old groups’ from the first trial (2005–2007) plus 648 ‘new groups’ implemented during the second trial (2009–2011). Groups convened monthly and worked through a learning and action cycle that involved group members identifying and prioritising issues affecting the health of mothers and newborns (Cycle 1) or women and children (Cycle 2) in their communities, and developing and implementing strategies to address these issues. New groups worked through Cycle 1, whereas old groups who had previously completed Cycle 1 during the first trial worked through Cycle 2. Groups were participatory, rather than prescriptive, ensuring strategies were culturally appropriate. Strategies differed across groups and included home visits, using social dramas and picture card games to raise awareness of common health problems, prevention and treatment strategies, and collecting voluntary donations to community funds to facilitate care seeking. Mental health issues were not explicitly addressed during either cycle. Local women of reproductive age with at least some high school education were recruited to facilitate group meetings, and groups held community meetings to encourage the wider community to engage with their strategies. Groups took approximately 20 months to work through the cycles. Both intervention and control clusters received health strengthening activities, including training of traditional birth attendants in essential newborn and maternal care, essential obstetrics and neonatology education sessions for doctors and provision of health equipment for community clinics.

### Target group and respondents

Women’s groups were open to all community members, however the target group was women of reproductive age, especially pregnant and newly married women. The women’s group intervention was expected to have effects reaching beyond group members to non-attenders. We therefore collected data at a population level from all women permanently residing in the study districts who gave birth during the trial period. We excluded women who were not permanently residing in the study area, as it was unlikely they were exposed to the women’s group intervention.

### Data collection and measurement of postpartum psychological distress

Traditional birth attendants identified all deaths and births in the study districts and received a financial incentive for reporting each event. Monitors visited the households to verify the birth or death. After a birth an interview was conducted between a minimum of six weeks and a maximum of 52 weeks postpartum. The interview included questions about healthcare, socioeconomic status and postpartum psychological distress. As in several community-based randomised controlled trials of participatory interventions, monitors and respondents were aware of allocation, however there were no incentives or disincentives for over- or under-reporting births, deaths or postpartum psychological distress. Furthermore, mechanisms existed to identify any inaccuracies, such as monitoring process data on a monthly basis and cross-checking a sample of data through re-interview [21].

Postpartum psychological distress was measured using the 20-item Self Reporting Questionnaire (SRQ-20) [24]. This tool has been used to screen for common mental disorders, including PCMDs, in a variety of low-income settings [25]. The tool comprises 20 yes/no questions about symptoms of depression and anxiety experienced in the past 30 days. Responses were summed to produce a total score ranging from 0 to 20.

The main trial sample size calculations were carried out for neonatal mortality. We also sought to estimate the power of the study to detect changes in postpartum psychological distress among mothers. We carried out power calculations using a cut-off SRQ score ≥6 to indicate the presence of distress for baseline prevalences ranging between 10–20%, with an estimated k value of 0.3 [26], [27]. Assuming 500 births per year per cluster, if the baseline prevalence of postpartum psychological distress was 10%, collecting data over 12 months would allow us to detect a 35% reduction in distress with 64.4–70.2% power at the 95% confidence level, and between 69 and 75% power if the baseline prevalence was 20%. Collecting data for 24 months would only yield a marginal increase in power (68.7–74.4%). In order not to impose a burden of continuous data collection on the monitoring team, we therefore planned to collect data on postpartum psychological distress for four months (January to April) per year over three years. We screened each mother for postpartum psychological distress once per birth: data are therefore cross-sectional. We did not exclude any mothers for mental ill-health reasons. Some mothers delivered more than once in the study period, because of multiple births (twins or triplets) or through repeated pregnancies. To avoid duplicating cases we therefore only used data associated with the firstborn infant of a multiple birth, or the first birth during the SRQ-20 data collection period.

### Statistical analysis

A researcher who was not involved in the design or management of the trial (KC) carried out the analysis independently. We assigned mothers to the cluster in which their delivery was registered, and estimated the intra-cluster correlation coefficient (ICC) for postpartum psychological distress using a large one-way ANOVA to assess how symptoms clustered. We used an SRQ-20 score ≥6 to report the prevalence of psychological distress because a previous study in urban Bangladesh found that this score discriminated best for psychiatric disorders, though the sensitivity (62%) and specificity (69%) were quite low [28]. In order to avoid over-reliance on this cut-off score, we therefore evaluated the impact of women’s groups using the SRQ-20 data as a continuous outcome, through a t-test of cluster means weighted by inverse variance, accounting for clustering at the union and district level [27]. We log transformed SRQ-20 data, adding 0.1 to each value to account for scores of zero. The results are reported on the original scale, interpreted as the ratio of the geometric mean SRQ-20 score in the intervention arm over the geometric mean score in the control arm. Analyses were not adjusted for confounders such as age, education or asset ownership, since these factors were relatively balanced across trial arms at baseline. We excluded a small number of cases with missing SRQ-20 data (0.4% 23/6275). We decided a priori to conduct analyses with and without tea garden residents. The trial in eastern India only reported a significant effect of women’s groups on distress in its final year [15]. We therefore planned to stratify our analyses by year of birth. Our main analyses used data collected during the trial period in 2010 and 2011. After the trial, groups carried on meeting and surveillance and data collection continued. Systematic differences in neonatal mortality rates between control and intervention arms were only evident from April 2011. If an intervention effect of women’s groups on postpartum psychological distress was dependent on a reduction in neonatal mortality, we would have been unable to detect it using data from the trial period only, since SRQ-20 data were collected only during the months of January to April. We therefore conducted a post-hoc sensitivity analysis including further SRQ-20 data collected between 1 January and 30 April 2012. All analyses were carried out in Stata version 12 [29]. We will make an anonymised version of the dataset used for the analyses in this article available to researchers upon completion of a simple data request form, which can be obtained from Dr Audrey Prost (Audrey.Prost@ucl.ac.uk).

## Results

**Figure 1:**
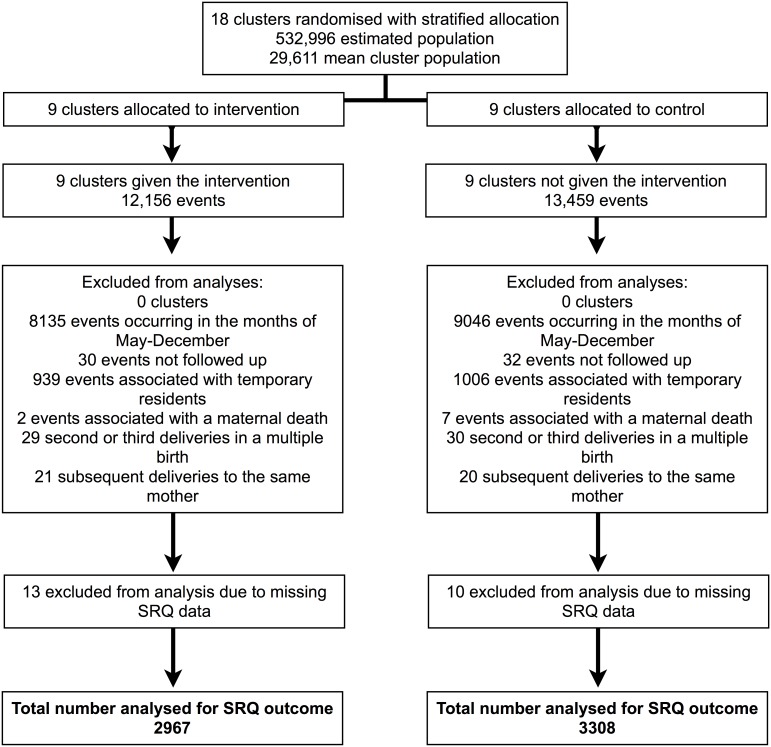
Sample selection procedure for evaluation of the effect of women’s groups on postpartum psychological distress. From 25,615 births and deaths (‘events’) over the 24 study months in 2010 and 2011 we excluded data from: 17,181 events that did not occur within the SRQ-20 data collection periods; 62 events where it was not possible to conduct an interview due to migration or refusal; 1945 events associated with mothers who were temporary residents in the study area; nine events associated with a maternal death; 100 events associated with mothers who had previously delivered during the SRQ-20 data collection periods, either because of multiple births or through repeated births; 23 mothers due to missing SRQ-20 data. In total, 6275 mothers were included in the final sample.

Figure 1 shows the selection process of data included in the trial analysis for the postpartum psychological distress outcome. In total, data were available for 25,615 births and deaths over the 24 study months in 2010 and 2011. After excluding data from events that did not occur within the SRQ-20 data collection periods (January to April inclusive, 2010 and 2011), there were 4021 and 4413 events in intervention and control clusters, respectively. Of these we excluded 62 events where it was not possible to conduct an interview due to migration or refusal. Although the exact number or mothers who refused interview is not known it is believed to be extremely low (<1%). We excluded data from 1945 events associated with mothers who were temporary residents in the study area and nine events associated with a maternal death. In order to remove data from duplicate interviews associated with the same mother, we excluded data from 100 events associated with mothers who had previously delivered during the SRQ-20 data collection period, either because of multiple births or through repeated births. At this stage the denominator for analyses was mothers rather than events and we excluded 23 mothers due to missing SRQ-20 data. In total, 6275 mothers were included in the final sample. The median interval between childbirth and interview was 70 days (interquartile range: 52–102) and it was similar in control and intervention areas.

### Mothers’ characteristics and women’s group participation

Table 1 shows that mothers in intervention and control arms were similar with respect to baseline characteristics. Overall, the average age of mothers was 25 years. Most were Muslim and a quarter had never been to school. Around a third had three or more household assets such as a radio, television, bicycle or mobile phone. Only a fifth delivered in facilities and most did not receive adequate antenatal healthcare. The socioeconomic status of respondents excluding the tea garden residents was higher.

**Table 1:** Respondent characteristics for intervention and control areas (with and without tea garden residents) at baseline and during the SRQ-20 data collection period.

	BASELINE (JANUARY–DECEMBER 2008)			SRQ-20 DATA COLLECTION (JAN–APRIL 2010 & 2011)		
	Intervention	Control	Control	Intervention	Control	Control
	(N = 5027)	Excludingtea-gardenresidents(N = 5013)	Includingtea-gardenresidents(N = 5571)	(N = 2967)	Excludingtea gardenresidents(N = 2823)	Includingtea gardenresidents(N = 3308)
**Age (years)**						
Mean (SD)	24.7 (5.6)	24.6 (5.5)	24.6 (5.4)	24.5 (5.3)	24.8 (5.4)	24.8 (5.4)
**Age at first** **pregnancy (years)**						
Mean (SD)	18.4 (2.8)	18.5 (2.7)	18.6 (2.6)	18.4 (2.6)	18.6 (2.6)	18.7 (2.6)
**Gravidity**						
Mean (SD)	2.7 (1.7)	2.5 (1.6)	2.5 (1.6)	2.5 (1.6)	2.5 (1.6)	2.5 (1.6)
**Religion**						
Islam (%)	4539 (90.3)	4523 (90.2)	4605 (82.7)	2626 (88.5)	2551 (90.4)	2594 (78.5)
Hindu (%)	479 (9.5)	487 (9.7)	959 (17.2)	341 (11.5)	270 (9.6)	708 (21.4)
Other (%)	9 (0.2)	3 (0.1)	7 (0.1)	0 (0)	1 (0.0)	1 (0.0)
**Education**						
Never went to school (%)	1261 (25.1)	1091 (21.8)	1437 (25.8)	617 (20.8)	529 (18.7)	810 (24.5)
Primary education (%)	1795 (35.7)	1609 (32.1)	1727 (31.0)	1129 (38.1)	942 (33.4)	1059 (32.0)
Secondary or above (%)	1971 (39.2)	2313 (46.1)	2407 (43.2)	1221 (41.2)	1352 (47.9)	1439 (43.5)
**Household assets** **						
None (%)	1840 (36.6)	1635 (32.6)	1939 (34.8)	630 (21.2)	534 (18.9)	737 (22.3)
One (%)	1056 (21.0)	1017 (20.3)	1087 (19.5)	802 (27.0)	708 (25.1)	801 (24.1)
Two (%)	722 (14.4)	636 (12.7)	697 (12.5)	438 (14.8)	449 (15.9)	511 (15.5)
Three or more (%)	1409 (28.0)	1723 (34.4)	1846 (33.1)	1097 (37.0)	1132 (40.1)	1259 (38.1)
**Perinatal health** **care utilization**						
Facility deliveries (%)	943 (18.8)	1065 (21.3)	1114 (20.0)	820 (27.6)	796 (28.2)	862 (26.1)
4 or more ANCcheck-ups by formalprovider (%)	546 (10.9)	648 (12.9)	676 (12.1)	541 (18.2)	386 (13.7)	398 (12.0)
**Counts of births** **and deaths**						
Number of births	4965	4930	5485	2876	2749	3216
Neonatal mortalityrate per 1000 livebirths	38.3	35.3	37.2	14.6	28.0	28.3

**Assets included in the variable: radio, electric fan, television, fridge, mobile phone, bicycle, generator and electricity.

Table 1 also shows that the sample of mothers screened with the SRQ-20 was similar to the full baseline sample in terms of socioeconomic characteristics. During the trial period, among the sample screened with the SRQ-20, more mothers received antenatal care in the intervention arm compared to the control arm and the neonatal mortality rate was lower in the intervention arm. These effects were probably due to the women’s group intervention. Otherwise, characteristics were balanced across trial arms. Table 2 shows rates of participation in women’s group activities for different subgroups of the study sample in intervention clusters. Participation was marginally lower among mothers living in the poorest households, as well as those who were primigravid, younger, or had a secondary education or above. The percentage of respondents who attended at least one group meeting was 33% (490/1507) in 2010, rising to 37% (547/1460) in 2011. Among mothers who had attended meetings, 83% (860/1037) attended on a monthly basis.

**Table 2:** Women’s group participation rates among respondents screened with the SRQ-20.

	Proportion ofwomen in interventionclusters (%) N = 2967	Proportion of women inintervention clusters thatattended groups (%) N = 1037
**Age group**		
<20 years	505 (17.0)	137 (13.2)
20–29 years	1889 (63.7)	676 (65.2)
30–39 years	549 (18.5)	220 (21.2)
40 years or older	24 (0.8)	4 (0.4)
**Religion**		
Muslim	2626 (88.5)	933 (90.0)
Hindu	341 (11.5)	104 (10.0)
Other	0 (0)	0 (0)
**Maternal education**		
Never went to school/less than 1 year	617 (20.8)	231 (22.3)
Primary education/non-formal	1129 (38.1)	441 (42.5)
Secondary or above	1221 (41.2)	365 (35.2)
**Household**		
None (%)	630 (21.2)	381 (19.7)
One (%)	802 (27.0)	537 (27.8)
Two (%)	438 (14.8)	267 (13.8)
Three or more (%)	1097 (37.0)	745 (38.6)
**Primigravid**	996 (32.6)	253 (24.0)
**Experienced a neonatal death or stillbirth**	133 (4.5)	53 (5.1)
**SRQ-20 score >6**	1028 (34.7)	351 (33.9)

### Impact of the women’s groups on postpartum psychological distress

Using a cut-off score of ≥6, the overall prevalence of postpartum psychological distress was 35% (2208/6275) (Table 3). Prevalence and mean SRQ-20 score were lower in the intervention arm, and between 2010 and 2011 there were substantial reductions in prevalence and mean SRQ-20 score in both intervention and control arms. Prevalence was highest in Moulvibazar, especially in 2010. The ICC for SRQ-20 scores was 0.10 (95% CI 0.04, 0.17), suggesting that mothers’ SRQ-20 scores within the same cluster were more similar to mothers’ scores across clusters. A weighted t-test of log transformed cluster mean SRQ-20 scores showed that overall there was no significant difference in mean SRQ-20 scores between intervention and control arms (β 1.44 95% CI 0.28, 3.08) (Table 4). Similarly we found no effect of the intervention by year, or when we excluded tea garden residents. We conducted a sensitivity analysis using data from January to April 2012 in order to assess the impact of women’s groups on postpartum psychological distress after a difference in neonatal mortality rates between trial arms had become apparent. We found no significant impact of the groups when these data were included in the analysis (including tea garden residents: β 1.28 95% CI 0.24, 2.91 P = 0.680; excluding tea garden residents: β 1.23 95% CI 0.23, 2.86 P = 0.735) or analysed separately (including tea garden residents: β 0.74 95% CI 0.15, 2.94 P = 0.662; excluding tea garden residents: β 0.70 95% CI 0.14, 2.83 P = 0.617).

**Table 3:** Summary measures of postpartum psychological distress (SRQ-20 score ≥6) in intervention and control areas (with and without tea garden residents), by year.

	Mothers(N)	Overallprevalence	District prevalence			Meanclusterscore	Meanclusterprevalence	Mean(SD)	Median(IQR)
			Bogra	Faridpur	Moulvibazar				
**2010 and 2011**									
Intervention	2967	1028 (34.7)	320 (34.7)	296 (27.9)	412 (41.9)	5.2 (1.8)	34.8 (16.8)	5.2 (4.3)	4 (2–8)
Control excluding teagarden residents	2823	1014 (35.9)	286 (30.2)	381 (34.3)	347 (45.4)	5.3 (1.4)	33.7 (14.0)	5.5 (4.5)	4 (2–9)
Control including teagarden residents	3308	1180 (35.7)	286 (30.2)	381 (34.3)	513 (41.1)	5.3 (1.2)	34.3 (12.2)	5.4 (4.6)	4 (2–8)
**2010, 2011 and 2012**									
Intervention	4260	1354 (31.8)	437 (32.8)	378 (25.4)	539 (37.5)	4.9 (1.7)	32.1 (16.3)	4.9 (4.1)	4 (2–8)
Control excluding teagarden residents	4072	1420 (34.9)	464 (33.5)	484 (30.5)	472 (42.8)	5.2 (1.4)	33.1 (15.0)	5.4 (4.5)	4 (2–8)
Control including teagarden residents	4760	1631 (34.3)	464 (33.5)	484 (30.5)	683 (38.1)	5.2 (1.3)	33.4 (13.1)	5.3 (4.5)	4 (2–8)
**2010**									
Intervention	1507	624 (41.4)	178 (39.7)	183 (33.7)	263 (51.0)	5.9 (1.8)	41.3 (15.7)	5.9 (4.6)	5 (2–9)
Control excluding teagarden residents	1389	566 (40.8)	143 (32.2)	219 (39.8)	204 (51.7)	5.7 (1.5)	38.5 (15.2)	5.9 (4.6)	5 (2–9)
Control including teagarden residents	1644	647 (39.4)	143 (32.2)	219 (39.8)	285 (43.9)	5.7 (1.3)	38.0 (12.7)	5.8 (4.7)	5 (2–9)
**2011**									
Intervention	1460	404 (27.7)	142 (30.0)	113 (21.8)	149 (31.8)	4.5 (2.2)	28.1 (21.4)	4.5 (3.9)	4 (1–7)
Control excluding teagarden residents	1434	448 (31.2)	143 (28.4)	162 (28.8)	143 (38.8)	4.8 (1.5)	30.3 (14.4)	5.1 (4.4)	4 (2–8)
Control including teagarden residents	1664	533 (32.0)	143 (28.4)	162 (28.8)	228 (38.1)	4.9 (1.3)	30.3 (14.4)	5.1 (4.4)	4 (1.5, 8)
**2012**									
Intervention	1293	326 (25.2)	117 (28.5)	82 (19.1)	127 (28.1)	4.3 (1.7)	25.6 (16.3)	4.2 (3.6)	3 (1–7)
Control excluding teagarden residents	1249	406 (32.5)	178 (40.7)	103 (21.8)	125 (36.9)	5.1 (2.0)	31.7 (20.8)	5.2 (4.2)	4 (2–8)
Control including teagarden residents	1452	451 (31.1)	178 (40.7)	103 (21.8)	170 (31.4)	5.0 (1.9)	31.2 (19.8)	5.0 (4.3)	4 (2–8)

**Table 4:** Evaluation of the effect of participatory women’s groups on postpartum psychological distress.

	Including tea garden residents			Excluding tea garden residents		
	Ratio of means	95% CI	P-value	Ratio of means	95% CI	P-value
**2010**	1.41	0.39, 3.53	0.509	1.14	0.35, 3.20	0.801
**2011**	1.78	0.17, 3.18	0.391	1.89	0.18, 3.37	0.350
**2012**	0.74	0.15, 2.94	0.662	0.70	0.14, 2.83	0.617
**2010 and 2011**	1.44	0.28, 3.08	0.524	1.38	0.28, 3.02	0.586
**2010, 2011 and 2012**	1.28	0.24, 2.91	0.680	1.23	0.23, 2.86	0.735

## Discussion

Consistent with previous studies, we report a high prevalence of postpartum psychological distress among mothers in rural Bangladesh [18], [19]. However, in contrast to our expectations, a participatory intervention with women’s groups did not significantly reduce postpartum psychological distress in this setting This is surprising given that the intervention substantially reduced neonatal mortality, a risk factor for distress, and that a similar intervention in rural eastern India significantly reduced postpartum psychological distress. Furthermore, coverage of groups and participation among pregnant women in Bangladeshi groups was relatively high, and these factors have been shown to predict effectiveness, at least for neonatal and maternal mortality outcomes [13].

One possible explanation for the absence of a mental health effect is that neonatal mortality was less important as a predictor of postpartum psychological distress in Bangladesh compared to eastern India because the baseline neonatal mortality rate was lower and/or because factors not addressed by the women’s groups were more important for maternal mental health. For example, communities in rural Bangladesh are strongly patriarchal and factors associated with gender-based victimization, including domestic violence, marital relationship problems and lack of education, are known to be associated with PCMDs in this setting [19], [30], [31]. Also, communities in rural Bangladesh are exposed to flooding, storms and epidemics. These environmental stressors possibly contribute to psychological distress through effects on income and physical health [32]. The increased prevalence of postpartum psychological distress in Moulvibazar during 2010 may have been due to severe flooding in the district at this time.

Another possible explanation for the null result is that mechanisms other than a reduction in neonatal mortality account for the mental health benefits of women’s groups in eastern India, and these mechanisms were not fully realised in Bangladesh. Contextual factors, including gender and social equality, are likely to influence the way in which women’s groups engage with non-group members in their communities. In rural Bangladesh women are secluded and confined to the house, and although group members disseminated health information among non-group members, with benefits for maternal and child health, social barriers may have prevented them from engaging with women in a way that empowered them more generally, and reduced psychological distress. Further investigation is needed to understand how contextual factors influence strategies used by women’s groups, as is research to establish mechanisms for their effects on postpartum psychological distress in eastern India. In India it took until the third year for these effects to be detected. It remains possible that the small differences we observed between trial arms in Bangladesh would increase over time, with the benefits of group participation in reducing distress emerging later.

Our study suggests that women’s groups practising participatory learning and action focused on women’s, newborn and child health are not universally effective to improve maternal mental health. Evaluations of alternative approaches are urgently needed in Bangladesh and other low-income countries in order to address the lack of evidence-based interventions for PCMDs in these settings. Women’s groups are a cost-effective method to engage with disadvantaged women. One approach could therefore be to adapt existing groups by integrating mental health content into participatory learning and action cycles in order to improve awareness of the consequences and causes of PCMDs, and to develop appropriate strategies to address them. Content on context-specific predictors of PCMDs, such as domestic violence and environmental resilience could also be included. Another approach would be to deliver psychological therapy through women’s groups. In Uganda, Chile and China, trials of interventions involving group-based cognitive behaviour and interpersonal therapy reported benefits for general and postpartum depression [33]–[38]. Formative work is needed to assess the cultural acceptability and feasibility of this approach in other settings.

Alternatively interventions could be delivered individually. Evidence from middle-income countries suggests that preventive and treatment programmes for PCMDs delivered during home visits can be effective. For example, a cognitive behaviour intervention delivered by lady health workers in Pakistan and an intervention to improve the quality of the mother infant relationship in South Africa showed promising results for postpartum depression [39], [40]. However, individually delivered interventions may be more resource-intensive than group approaches and data are needed to assess the cost-effectiveness of these interventions.

### Study limitations

Our analysis has three main limitations. First, we only collected data between January and April in 2010, 2011 and 2012. The confidence intervals associated with results in Table 4 are wide, and collecting more data would have enabled greater precision. Also, in March and April, farm labourers may be unemployed after the *boro* crop has been planted and households may therefore be at risk of financial hardship and food insecurity [41]. During these months benefits of the women’s groups for maternal mental health may have been masked. Second, we did not assess inter-rater reliability for the SRQ-20, and therefore differences in mean cluster SRQ-20 scores could be related to inconsistencies between assessors. However, this is unlikely to have resulted in systematic bias since there were many assessors working in multiple unions, in both control and intervention arms. Intervention and surveillance teams were aware of cluster allocation, though there were no incentives for under or over reporting psychological distress. Finally, although we screened most mothers for distress in the first few weeks after childbirth, some were screened up to a year after delivery and we were unable to investigate differential effects of the intervention on early versus late postpartum psychological distress. Because the women’s group intervention was designed to benefit individuals beyond those attending groups, we assessed its impact at the population level and did not conduct subanalyses of individuals in the intervention arm.

### Conclusions

Participatory women’s groups in rural Bangladesh did not reduce postpartum psychological distress, possibly due to contextual factors and the relative importance of neonatal mortality as a population-level predictor of distress in this setting. An investigation of how contextual factors affect the mechanisms of women’s groups across settings is needed, as is local, formative research to identify ways of adapting group-based or individually delivered interventions for maternal mental health.

## References

[1] Goldberg D, Huxley P (1992) Common mental disorders: a biosocial model. London, UK: Tavistock/Routledge.

[2] Harris EC, Barraclough B (1997) Suicide as an outcome for mental disorders: a meta-analysis. British Journal of Psychiatry 170: 205–228. 9229027 10.1192/bjp.170.3.205

[3] Prince M, Patel V, Saxena S, Maj M, Maselko J, et al (2007) No health without mental health. Lancet 370: 859–877. 17804063 10.1016/S0140-6736(07)61238-0

[4] Rahman A, Iqbal Z, Bunn J, Lovel H, Harrington R (2004) Impact of maternal depression on infant nutritional status and illness: a cohort study. Archives of General Psychiatry 61: 946–952. 15351773 10.1001/archpsyc.61.9.946

[5] Senturk V, Hanlon C, Medhin G, Dewey M, Araya M, et al (2012) Impact of perinatal somatic and common mental disorder symptoms on functioning in Ethiopian women: The P-MaMiE population-based cohort study. Journal of Affective Disorders 136: 340–349. 22196052 10.1016/j.jad.2011.11.028 PMC3314986

[6] Cooper PJ, Tomlinson M, Swartz L, Woolgar M, Murray L, et al (1999) Postpartum depression and the mother-infant relationship in a South African peri-urban settlement. British Journal of Psychiatry 175: 554–548. 10789353 10.1192/bjp.175.6.554

[7] Parsons CE, Young KS, Rochat TJ, Kringelbach ML, Stein A (2012) Postnatal depression and its effects on child development: a review of evidence from low- and middle-income countries. British Medical Bulletin 101: 57–79. 22130907 10.1093/bmb/ldr047

[8] Surkan PJ, Kennedy CE, Hurley KM, Black MM (2011) Maternal depression and early childhood growth in developing countries: systematic review and meta-analysis. Bulletin of the World Health Organization 89. 10.2471/BLT.11.088187 PMC3150769 21836759

[9] Fisher J, Mello CD, Patel V, Rahman A, Tran T, et al (2012) Prevalence and determinants of common perinatal mental disorders in women in low- and lower-middle- income countries: a systematic review. Bulletin of the World Health Organisation 90: 139–149. 10.2471/BLT.11.091850 PMC3302553 22423165

[10] Clarke K, King M, Prost A (2013) Psychosocial Interventions for Perinatal Common Mental Disorders Delivered by Non-Mental Health Specialists in Low and Middle-Income Countries: A Systematic Review and Meta-Analysis. PLoS Medicine 10: e100154. 10.1371/journal.pmed.1001541 PMC3812075 24204215

[11] Rahman A, Fisher J, Bower P, Luchters S, Tran T, et al (2013) Interventions for common perinatal mental disorders in women in low- and middle-income countries: a systematic review and meta-analysis. Bulletin of the World Health Organization. 10.2471/BLT.12.109819 PMC3738304 23940407

[12] Hanlon C (2012) Maternal depression in low- and middle- income countries. International Health 5: 4–5. 24029837 10.1093/inthealth/ihs003

[13] Prost A, Colbourn T, Seward N, Azad K, Coomarasamy A, et al (2013) Women's groups practising participatory learning and action to improve maternal and newborn health in low-resource settings: a systematic review and meta-analysis. Lancet 381: 1736–1746. 23683640 10.1016/S0140-6736(13)60685-6 PMC3797417

[14] Rath S, Nair N, Tripathy P, Barnett S, Rath S, et al. (2010) Explaining the impact of a women's group led community mobilisation intervention on maternal and newborn health outcomes: the Ekjut trial process evaluation. BMC Health and Human Rights 10. 10.1186/1472-698X-10-25 PMC2987759 20969787

[15] Tripathy P, Nair N, Barnett S, Mahapatra R, Borghi J, et al (2010) Effect of a participatory intervention with women's groups on birth outcomes and maternal depression in Jharkhand and Orissa, India: a cluster-randomised controlled trial. Lancet 375: 1182–1192. 20207411 10.1016/S0140-6736(09)62042-0

[16] Azad K, Barnett S, Banerjee B, Shaha S, Khan K, et al (2010) Effect of scaling up women's groups on birth outcomes in three rural districts in Bangladesh: a cluster-randomised controlled trial. Lancet 375: 1193–1202. 20207412 10.1016/S0140-6736(10)60142-0

[17] Fottrell E, Azad K, Kuddus A, Younes L, Shaha S, et al (2013) The effect of increased coverage of participatory women's groups on neonatal mortality in Bangladesh: a cluster randomized trial. JAMA Pediatrics 20: 1–9. 10.1001/jamapediatrics.2013.2534 PMC5082727 23689475

[18] Black MM, Baqui AH, Zaman K, McNary SW, Le K, et al (2007) Depressive symptoms among rural Bangladeshi mothers: implications for infant development. Journal of Child Psychology and Psychiatry 48: 764–772. 17683448 10.1111/j.1469-7610.2007.01752.x

[19] Gausia K, Fisher C, Ali M, Oosthuizen J (2009) Magnitude and contributory factors of postnatal depression: a community-based cohort study from a rural subdistrict of Bangladesh. Psychological Medicine 39: 999–1007. 18812008 10.1017/S0033291708004455

[20] World Health Organisation (2007) WHO-AIMS report on mental health system in Bangladesh. Dhaka.

[21] Houweling TA, Azad K, Younes L, Kuddus A, Shaha S, et al (2011) The effect of participatory women's groups on birth outcomes in Bangladesh: does coverage matter? Study protocol for a randomized controlled trial. Trials 12: 208. 21943044 10.1186/1745-6215-12-208 PMC3197496

[22] Chowdhury M, Hasan G, Karim M (2011) A study on existing WATSAN condition in two twa gardens in Maulvibazar. Journal of Environmental Science and Natural Resources 4: 13–18.

[23] Ahmed M, Begum A, Chowdhury M (2010) Social constraints before sanitation improvement in tea gardens of Sylhet, Bangladesh. Environmental Monitoring and Assessment 164: 263–271. 19365608 10.1007/s10661-009-0890-0

[24] World Health Organization (1994) A user’s guide to self-reporting questionnaires. Geneva.

[25] Harpham T, Reichenheim M, Oser R, Thomas E, Hamid N, et al (2003) Measuring mental health in a cost-effective manner. Health Policy and Planning 18: 344–349. 12917276 10.1093/heapol/czg041

[26] Hayes RJ, Bennett S (1999) Simple sample size calculation for cluster-randomised trials. International Epidemiological Association 28: 319–326. 10.1093/ije/28.2.319 10342698

[27] Hayes RJ, Moulton LH (2009) Cluster randomised trials: Chapman & Hall/CRC Interdisciplinary Statistics.

[28] Islam MM, Ali M, Ferroni P, Underwood P, Alam MF (2000) Validity of a self reporting questionnaire (SRQ) in detecting psychiatric illnesses in an Uraban [sic] community in Bangladesh. Bangladesh Journal of Psychiatry 14: 31–43.

[29] StataCorp (2011) Stata Statistical Software: Release 12. In: College Station, editor. TX. StataCorp LP,.

[30] Gausia K, Fisher C, Ali M, Oosthuizen J (2009) Antenatal depression and suicidal ideation among rural Bangladeshi women: a community-based study. Archives of Women's Mental Health 12: 351–358. 10.1007/s00737-009-0080-7 19468825

[31] Nasreen HE, Kabir ZN, Forsell Y, Edhborg M (2011) Prevalence and associated factors of depressive and anxiety symptoms during pregnancy: a population based study in rural Bangladesh. BMC Women's Health 11. 10.1186/1472-6874-11-22 PMC3117808 21635722

[32] Choudhury WA, Quraishi FA, Haque Z (2006) Mental health and psychosocial aspects of disaster preparedness in Bangladesh. International Review of Psychiatry 18: 529–535. 17162693 10.1080/09540260601037896

[33] Araya R, Rojas G, Fritsch R, Gaete J, Rojas M, et al (2003) Treating depression in primary care in low-income women in Santiago, Chile: a randomised controlled trial. Lancet 361: 995–1000. 12660056 10.1016/S0140-6736(03)12825-5

[34] Bolton P, Bass J, Betancourt T, Speelman L, Onyango G, et al (2007) Interventions for depression symptoms among adolescent survivors of war and displacement in northern Uganda. Journal of the American Medical Association 298: 519–527. 17666672 10.1001/jama.298.5.519

[35] Bolton P, Bass J, Neugebauer R, Verdeli H, Clougherty KF, et al (2003) Group interpersonal psychotherapy for depression in rural Uganda: a randomized controlled trial. Journal of the American Medical Association 289: 3117–3124. 12813117 10.1001/jama.289.23.3117

[36] Gao L, Chan SW, Li X, Chen S, Hao Y (2010) Evaluation of an interpersonal psychotherapy-oriented childbirth education programme for Chinese first-time childbearing women: a randomised controlled trial. International Journal of Nursing Studies 47: 1208–1216. 20362992 10.1016/j.ijnurstu.2010.03.002

[37] Mao H-J, Li H-J, Chiu H, Chan W-C, Chen S-L (2012) Effectiveness of antenatal emotional self-management training program in prevention of postnatal depression in Chinese women. Perspectives in Psychiatric Care 48: 218–224. 23005589 10.1111/j.1744-6163.2012.00331.x

[38] Rojas G, Fritsch R, Solis J, Jadresic E, Castillo C, et al (2007) Treatment of postnatal depression in low-income mothers in primary-care clinics in Santiago, Chile: a randomised controlled trial. Lancet 370: 1629–1637. 17993363 10.1016/S0140-6736(07)61685-7

[39] Cooper PJ, Tomlinson M, Swartz L, Landman M, Molteno C, et al (2009) Improving quality of mother-infant relationship and infant attachment in socioeconomically deprived community in South Africa: randomised controlled trial. British Medical Journal 338: 997. 10.1136/bmj.b974 PMC2669116 19366752

[40] Rahman A, Malik A, Sikander S, Roberts C, Creed F (2008) Cognitive behaviour therapy-based intervention by community health workers for mothers with depression and their infants in rural Pakistan: a cluster-randomised controlled trial. Lancet 372: 902–909. 18790313 10.1016/S0140-6736(08)61400-2 PMC2603063

[41] Zug S (2006) Monga - Seasonal food insecurity in Bangladesh - Bringing the information together. Journal of Social Studies 11.

