# Correction: Impact of a Participatory Intervention with Women's Groups on Psychological Distress among Mothers in Rural Bangladesh: Secondary Analysis of a Cluster-Randomised Controlled Trial

**DOI:** 10.1371/journal.pone.0183203

**Published:** 2017-08-10

**Authors:** Kelly Clarke, Kishwar Azad, Abdul Kuddus, Sanjit Shaha, Tasmin Nahar, Bedowra Haq Aumon, Mohammed Munir Hossen, James Beard, Anthony Costello, Tanja A. J. Houweling, Audrey Prost, Edward Fottrell

There is an error in Fig 1. Please see the complete, corrected Fig 1 here.

**Fig 1:**
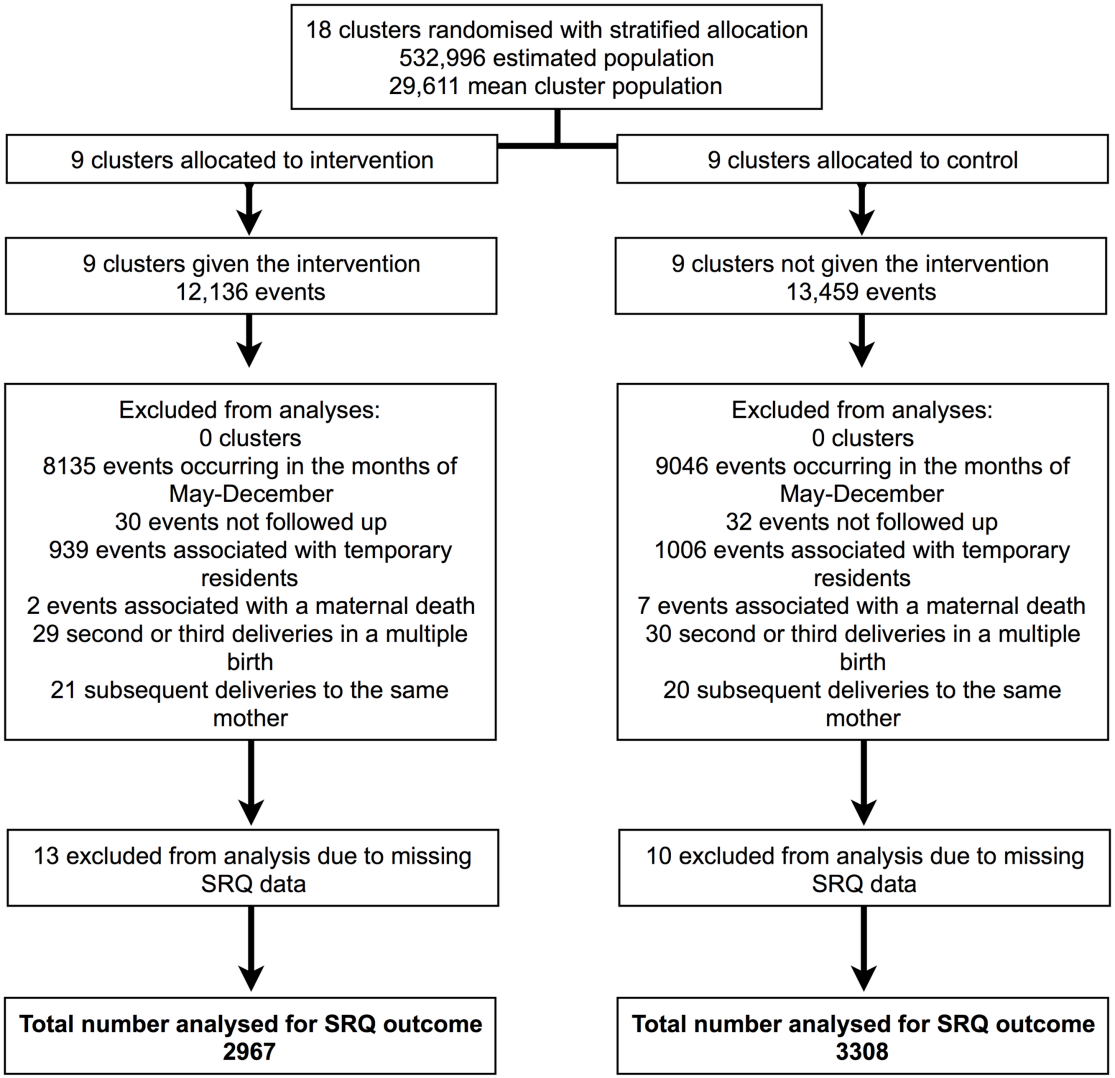
Sample selection procedure for evaluation of the effect of women’s groups on postpartum psychological distress. From 25,595 births and deaths (‘events’) over the 24 study months in 2010 and 2011 we excluded data from: 17,181 events that did not occur within the SRQ-20 data collection periods; 62 events where it was not possible to conduct an interview due to migration or refusal; 1945 events associated with mothers who were temporary residents in the study area; nine events associated with a maternal death; 100 events associated with mothers who had previously delivered during the SRQ-20 data collection periods, either because of multiple births or through repeated births; 23 mothers due to missing SRQ-20 data. In total, 6275 mothers were included in the final sample.

There are errors in Table 1. Please see the complete, correct Table 1 here.

**Table 1:** Respondent characteristics for intervention and control areas (with and without tea garden residents) at baseline and during the SRQ-20 data collection period.

	BASELINE (JANUARY—DECEMBER 2008)			SRQ-20 DATA COLLECTION (JAN—APRIL 2010 & 2011)		
	Intervention	Control	Control	Intervention	Control	Control
	(N = 5027)	Excluding tea-garden residents (N = 5013)	Including tea-garden residents (N = 5571)	(N = 2967)	Excluding tea garden residents (N = 2823)	Including tea garden residents (N = 3308)
**Age (years)**						
Mean (SD)	24.7 (5.6)	24.6 (5.4)	24.6 (5.4)	24.5 (5.3)	24.8 (5.4)	24.8 (5.4)
**Age at first pregnancy (years)**						
Mean (SD)	18.4 (2.8)	18.5 (2.7)	18.6 (2.6)	18.4 (2.6)	18.6 (2.6)	18.7 (2.6)
**Gravidity**						
Mean (SD)	2.7 (1.7)	2.5 (1.6)	2.5 (1.6)	2.5 (1.6)	2.5 (1.6)	2.5 (1.6)
**Religion**						
Islam (%)	4539 (90.3)	4523 (90.2)	4605 (82.7)	2626 (88.5)	2551 (90.4)	2594 (78.5)
Hindu (%)	479 (9.5)	487 (9.7)	959 (17.2)	341 (11.5)	270 (9.6)	708 (21.4)
Other (%)	9 (0.2)	3 (0.1)	7 (0.1)	0 (0)	1 (0.0)	1 (0.0)
**Education**						
Never went to school (%)	1261 (25.1)	1091 (21.8)	1437 (25.8)	617 (20.8)	529 (18.7)	810 (24.5)
Primary education (%)	1795 (35.7)	1609 (32.1)	1727 (31.0)	1129 (38.1)	942 (33.4)	1059 (32.0)
Secondary or above (%)	1971 (39.2)	2313 (46.1)	2407 (43.2)	1221 (41.2)	1352 (47.9)	1439 (43.5)
Household assets**						
None (%)	1840 (36.6)	1635 (32.6)	1939 (34.8)	630 (21.2)	534 (18.9)	737 (22.3)
One (%)	1056 (21.0)	1017 (20.3)	1087 (19.5)	802 (27.0)	708 (25.1)	801 (24.2)
Two (%)	722 (14.4)	636 (12.7)	697 (12.5)	438 (14.8)	449 (15.9)	511 (15.5)
Three or more (%)	1409 (28.0)	1723 (34.4)	1846 (33.2)	1097 (37.0)	1132 (40.1)	1259 (38.1)
**Perinatal health care utilization**						
Facility deliveries (%)	943 (18.8)	1065 (21.3)	1114 (20.0)	820 (27.6)	796 (28.2)	862 (26.1)
4 or more ANC check-ups by formal provider (%)	546 (10.9)	648 (12.9)	676 (12.1)	541 (18.2)	386 (13.7)	398 (12.0)
**Counts of births and deaths**						
Number of births	4965	4930	5485	2876	2749	3216
Neonatal mortality rate per 1000 livebirths	38.3	35.3	37.2	14.6	28.0	28.3

** Assets included in the variable: radio, electric fan, television, fridge, mobile phone, bicycle, generator and electricity

There are errors in Table 2. Please see the complete, correct Table 2 here.

**Table 2:** Women’s group participation rates among respondents screened with the SRQ-20.

	Proportion of women in intervention clusters (%) N = 2967	Proportion of women in intervention clusters that attended groups (%) N = 1037
**Age group**		
< 20 years	505 (17.0)	137 (13.2)
20–29 years	1889 (63.7)	676 (65.2)
30–39 years	549 (18.5)	220 (21.2)
40 years or older	24 (0.8)	4 (0.4)
**Religion**		
Muslim	2626 (88.5)	933 (90.0)
Hindu	341 (11.5)	104 (10.0)
Other	0 (0)	0 (0)
**Maternal education**		
Never went to school/less than 1 year	617 (20.8)	231 (22.3)
Primary education/non-formal	1129 (38.1)	441 (42.5)
Secondary or above	1221 (41.2)	365 (35.2)
**Household assets**		
None (%)	630 (21.2)	249 (24.0)
One (%)	802 (27.0)	265 (25.6)
Two (%)	438 (14.8)	171 (16.5)
Three or more (%)	1097 (37.0)	352 (33.9)
**Primigravid**	966 (32.6)	253 (24.4)
**Experienced a neonatal death or stillbirth**	133 (4.5)	53 (5.1)
**SRQ-20 score >6**	1028 (34.7)	351 (33.9)

There are errors in Table 3. Please see the complete, correct Table 3 here.

**Table 3:** Summary measures of postpartum psychological distress (SRQ-20 score >6) in intervention and control areas (with and without tea garden residents), by year.

	Mothers (N)	Overall prevalence	District prevalence			Mean cluster score	Mean cluster prevalence	Mean (SD)	Median (IQR)
			Bogra	Faridpur	Moulvibazar				
**2010 and 2011**									
Intervention	2967	1028 (34.7)	320 (34.7)	296 (27.9)	412 (41.9)	5.2 (1.8)	34.8 (16.8)	5.2 (4.3)	4 (2–8)
Control excluding tea garden residents	2823	1014 (35.9)	286 (30.2)	381 (34.3)	347 (45.4)	5.3 (1.4)	33.7 (14.0)	5.5 (4.5)	4 (2–9)
Control including tea garden residents	3308	1180 (35.7)	286 (30.2)	381 (34.3)	513 (41.1)	5.3 (1.2)	34.3 (12.2)	5.4 (4.6)	4 (2–8)
**2010, 2011 and 2012**									
Intervention	4260	1354 (31.8)	437 (32.8)	378 (25.4)	539 (37.5)	4.9 (1.7)	32.1 (16.3)	4.9 (4.1)	4 (2–8)
Control excluding tea garden residents	4072	1420 (34.9)	464 (33.5)	484 (30.5)	472 (42.8)	5.2 (1.4)	33.1 (15.0)	5.4 (4.5)	4 (2–8)
Control including tea garden residents	4760	1631 (34.3)	464 (33.5)	484 (30.5)	683 (38.1)	5.2 (1.3)	33.4 (13.1)	5.3 (4.5)	4 (2–8)
**2010**									
Intervention	1507	624 (41.4)	178 (39.7)	183 (33.7)	263 (51.0)	5.9 (1.8)	41.3 (15.7)	5.9 (4.6)	5 (2–9)
Control excluding tea garden residents	1389	566 (40.8)	143 (32.2)	219 (39.8)	204 (51.7)	5.7 (1.5)	38.5 (15.2)	5.9 (4.6)	5 (2–9)
Control including tea garden residents	1644	647 (39.4)	143 (32.2)	219 (39.8)	285 (43.9)	5.7 (1.3)	38.0 (12.7)	5.8 (4.7)	5 (2–9)
**2011**									
Intervention	1460	404 (27.7)	142 (30.0)	113 (21.8)	149 (31.8)	4.5 (2.2)	28.1 (21.4)	4.5 (3.9)	4 (1–7)
Control excluding tea garden residents	1434	448 (31.2)	143 (28.4)	162 (28.8)	143 (38.8)	4.8 (1.5)	28.5 (15.1)	5.1 (4.4)	4 (2–8)
Control including tea garden residents	1664	533 (32.0)	143 (28.4)	162 (28.8)	228 (38.1)	4.9 (1.3)	30.3 (14.4)	5.1 (4.4)	4 (1.5–8)
**2012**									
Intervention	1293	326 (25.2)	117 (28.5)	82 (19.1)	127 (28.1)	4.3 (1.7)	25.6 (16.3)	4.2 (3.6)	3 (1–7)
Control excluding tea garden residents	1249	406 (32.5)	178 (40.7)	103 (21.8)	125 (36.9)	5.1 (2.0)	31.7 (20.8)	5.2 (4.3)	4 (2–8)
Control including tea garden residents	1452	451 (31.1)	178 (40.7)	103 (21.8)	170 (31.4)	5.0 (1.9)	31.2 (19.8)	5.0 (4.3)	4 (2–8)

There is an error in the third sentence of the Statistical Analysis subsection of the Methods. The correct sentence is: We used an SRQ-20 score >6 to report the prevalence of psychological distress because a previous study in urban Bangladesh found that this score discriminated best for psychiatric disorders, though the sensitivity (62%) and specificity (69%) were quite low [28].

There is an error in the second sentence of the Results section. The correct sentence is: In total, data were available for 25,595 births and deaths over the study months in 2010 and 2011.

There is an error in the first sentence of the Impact of the women’s groups on postpartum psychological distress section of the Results. The correct sentence is: Using a cut-off score of >6, the overall prevalence of postpartum psychological distress was 35% (2208/6275) (Table 3).
